# Cesarean Section Trends and Associated Factors at a Tertiary Care Center in India: A Retrospective Study

**DOI:** 10.7759/cureus.73308

**Published:** 2024-11-08

**Authors:** Najma Malik, Babita Kapoor, Roshani Singh, Ruma Sarkar, Imran Ahmed Khan

**Affiliations:** 1 Obstetrics and Gynecology, Baba Raghav Das Medical College, Gorakhpur, IND; 2 Obstetrics and Gynecology, Maharshi Devraha Baba Autonomous State Medical College, Deoria, IND; 3 Community Medicine, Baba Raghav Das Medical College, Gorakhpur, IND

**Keywords:** cesarean section, decision-making, maternal welfare, trends, vaginal delivery

## Abstract

Background

Cesarean section (CS) is one of the most common surgical procedures performed on women globally, and its prevalence has been rising significantly over the past few decades. CS rates have been increasing globally, raising public health concerns due to the associated financial burden and increased health risks compared to vaginal delivery.

Methodology

This study involves a retrospective analysis of delivery records from a tertiary care hospital in Uttar Pradesh, India, over 10 years, from January 2011 to December 2021.

Results

The data presented shows a significant shift in delivery methods over the past decade, particularly in the increasing rate of CS at our tertiary care center. From 2011 to 2021, the rate of CS increased from 39.6% to 52.4%. A detailed examination of the indications for primary elective CS reveals that oligohydramnios with intrauterine growth restriction (IUGR) remained the most common indication, increasing from 31% in 2011 to 34.3% in 2021. Other significant indications include malpresentation and cephalopelvic disproportion.

Conclusion

The study indicates a rising trend in CS rates at the tertiary care center, with various clinical and non-clinical factors influencing this increase. The increasing rates of maternal requests for CS, both primary and repeat, point towards a shift in patient preferences and expectations. Additionally, the changing trends in indications for both elective and emergency CS highlight evolving clinical practices and an increased emphasis on maternal and fetal safety. Continuous monitoring and analysis of these trends are essential to ensure that the indications for CS are appropriately managed, balancing the benefits and risks associated with surgical deliveries.

## Introduction

Cesarean section (CS) is one of the most common surgical procedures performed on women globally, and its proportion among modes of delivery has been rising significantly over the past few decades [1]. The World Health Organization (WHO) recommends that the population-based CS rate should not exceed 15% of all live births, yet many tertiary care centers in India report rates exceeding 30% [2]. Emergency CS was more common than elective, with the most frequent indication being previous CS [3,4]. CS rates have been increasing globally, raising public health concerns due to the associated financial burden and increased maternal health risks compared to vaginal delivery. Like all other surgical procedures, cesarean sections can have risks like hemorrhage, infections, slower recovery time, delayed establishment of breastfeeding, and increased likelihood of complications in future pregnancy. There is an increasing incidence of the morbid placenta which has a high mortality rate and other pregnancy-related complications leading to the increasing rate of CS rate [5,6]. Another study shows CS rates in India increased significantly between 2010 and 2017, with private facilities having 40% higher odds of having a CS compared to public facilities and rates having unequal spatial distributions [7].

Reducing unnecessary CS has become a significant focus in obstetric care due to the rise in CS deliveries and their associated public health implications. Reducing primary CS rates can decrease the likelihood of complications in subsequent pregnancies, such as increased blood loss, chances of infections, abnormal placentation, and uterine rupture [8]. Unnecessary CS can also place a strain on healthcare resources, increasing costs and increasing hospital stays. So, providing evidence-based maternity care to improve overall maternal and infant health outcomes is needed. With this background, this retrospective study was conducted. The primary objective of this study is to analyze trends in CS rates over a decade at a tertiary care center in India. Secondary objectives include evaluating the clinical and non-clinical factors contributing to the rising CS rates.

## Materials and methods

To study the CS trends and associated factors, data of all registered pregnant patients were collected from hospital delivery records. Data with incomplete records and those with <28 weeks gestation were excluded. All the deliveries (vaginal as well as cesarean) conducted between January 1 and December 31 in 2011, 2016, and 2021 in the Department of Obstetrics and Gynecology, Baba Raghav Das Medical College, Gorakhpur, the only referral center of eastern Uttar Pradesh, India, were taken. Data on vaginal deliveries as well as CS along with their indications were recorded. Primary, repeat, emergency, and elective CS were calculated for each year.

Ethical approval for the study was obtained from the Institutional Ethical Committee of Baba Raghav Das Medical College (approval number: 38/IHEC/2024) on May 4, 2024. Because secondary data analysis was carried out in the study, informed consent of the participants was not applicable. Anonymity and confidentiality of data were maintained.

## Results

A total of 2405, 3740, and 3630 live births occurred in the hospital in 2011, 2016, and 2021. The analysis revealed a significant increase in the CS rate from 39.6% in 2011 to nearly 52.4% in 2021.

A total of 9,775 deliveries were conducted in our tertiary center during our study period (2011, 2016, 2021). The rate of vaginal delivery is decreased over a decade from 60.37% in 2011 to 47.58% in 2021, whereas the rate of CS increased from 39.63% in 2011 to 52.42% in 2021 (Table 1).

**Table 1:** Mode of delivery over a decade from 2011 to 2021

Year	Total number of deliveries	Vaginal delivery		Cesarean section	
		n	%	n	%
2011	2405	1452	60.37	953	39.63
2016	3740	1952	52.19	1788	47.81
2021	3630	1727	47.58	1903	52.42
Total	9775	5131		4644	

**Figure 1:**
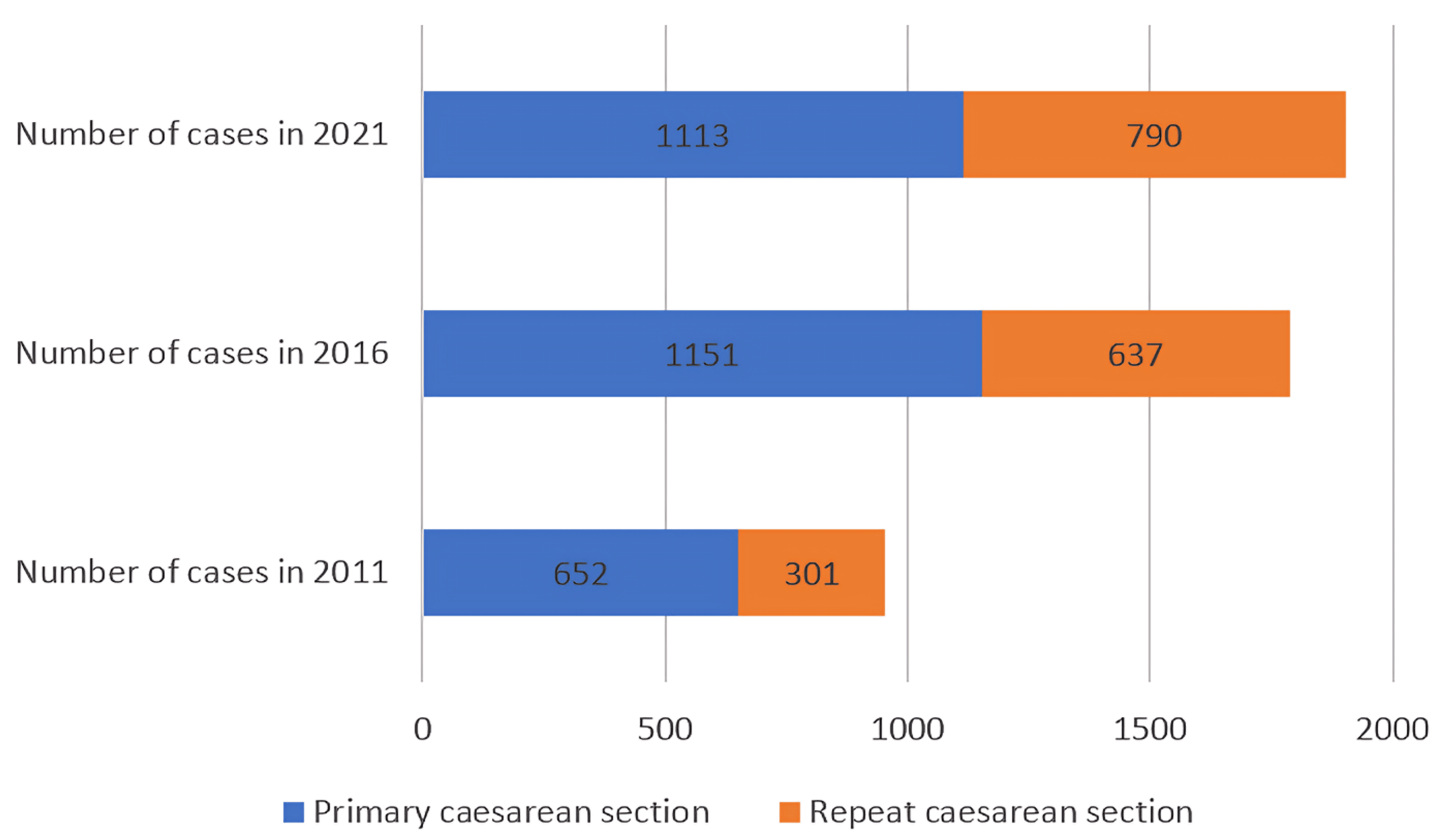
Type of cesarean section over a decade from 2011 to 2021

There is a decreasing trend seen in the primary CS rate, i.e., 68.42% in 2011, 64.37% in 2016, and 58.49% in 2021. An increasing trend is seen in repeat CS, 31.58%, 35.63%, and 41.51% in 2011, 2016, and 2021, respectively (Figure 1).

Table 2 shows indications for CS. The most common indication of CS was oligohydramnios with intrauterine growth restriction (IUGR) (30.97% in 2011, 33.33% in 2016, and 34.30% in 2021). Malpresentation was found to be the second most common indication for primary elective CS throughout the decade (26.54%, 21.05%, and 21.49%). Maternal request as an indication for performing primary elective CS shows an increasing trend from 6.19% in 2011 to 15.70% in 2021.

**Table 2:** Indications of primary elective cesarean sections IUGR: intrauterine growth retardation; CPD: cephalopelvic disproportion; APH: antepartum hemorrhage

Indications	Number of cases in 2011		Number of cases in 2016		Number of cases in 2021	
	n	%	n	%	n	%
Oligohydramnios with IUGR	35	30.97	38	33.33	83	34.30
Malpresentation	30	26.54	24	21.05	52	21.49
CPD	22	19.47	25	21.93	23	9.50
APH	19	16.81	17	14.91	46	19.01
Maternal request	7	6.19	10	8.77	38	15.70
Total number of cases	113		114		242	

The most common indication for primary CS over a decade is fetal distress (Table 3). Some other common indications are non-progress of labor, antepartum hemorrhage, and malpresentation. There is seen an increasing incidence of some indications over a decade like antepartum eclampsia from 1.48% (2011) to 14.93% (2021), severe pre-eclamptic toxemia from 1.86% (2011) to 10.10% (2021), and maternal request from 1.865 (2011) to 6.08% (2021).

**Table 3:** Indications of primary emergency cesarean sections NPOL: non-progress of labor; APH: antepartum hemorrhage; PET: pre-eclamptic toxemia; CPD: cephalopelvic disproportion

Indications	Number of cases in 2011		Number of cases in 2016		Number of cases in 2021	
	n	%	n	%	n	%
Fetal distress	154	28.57%	290	27.96%	152	17.45%
NPOL	82	11.13%	132	10.80%	162	18.60%
APH	80	14.84%	104	10.02%	75	8.61%
Malpresentation	66	12.24%	105	10.13%	78	8.96%
Severe PET	10	1.86%	65	6.27%	88	10.10%
Antepartum eclampsia	8	1.48%	120	11.57%	130	14.93%
Obstructed labor	71	13.17%	75	7.23%	61	7.00%
CPD	58	7.05%	95	6.75%	72	8.27%
Maternal request	10	1.86%	51	4.9%	53	6.08%
Total	539	100%	1037	100%	871	100%

The most common indication in the repeat elective CS group was oligohydramnios with IUGR 30.88%, 29.25%, and 51.63% in 2011, 2016, and 2021, respectively. There was an increase in the incidence of maternal requests as an indication over the decade from 5.88% in 2011 to 23.91% in 2021 (Table 4).

**Table 4:** Indications of repeat elective cesarean sections IUGR: intrauterine growth retardation; CPD: cephalopelvic disproportion; APH: antepartum hemorrhage

Indications	Number of cases in 2011		Number of cases in 2016		Number of cases in 2021	
	n	%	n	%	n	%
Oligohydramnios with IUGR	21	30.88%	31	29.25%	95	51.63%
CPD	19	27.94%	27	25.47%	23	12.5%
Malpresentation	12	17.65%	21	19.81%	9	4.89%
APH	12	17.65%	14	13.21%	13	7.07%
Maternal request	4	5.88%	13	12.26%	44	23.91%
Total	68	100%	106	100%	184	100%

The most common indications for repeat emergency CS are scar tenderness, fetal distress, non-progress of labor, and cephalopelvic disproportion which contributes the maximum throughout the study period (Table 5).

**Table 5:** Indications of repeat emergency cesarean sections NPOL: non-progress of labor; CPD: cephalopelvic disproportion; APH: antepartum hemorrhage; PET: pre-eclamptic toxemia

Indications	Number of cases in 2011		Number of cases in 2016		Number of cases in 2021	
	n	%	n	%	n	%
Scar tenderness	79	33.90%	243	45.76%	258	42.57%
Fetal distress	41	17.59%	103	19.40%	91	15.02%
NPOL	23	9.87%	58	10.92%	50	8.25%
CPD	23	9.87%	35	6.59%	69	11.39%
APH	22	9.44%	24	4.52%	9	1.49%
Malpresentation	13	5.58%	14	2.64%	30	4.95%
Obstructed labor	15	6.44%	12	2.26%	0	0%
Severe PET	8	3.43%	18	3.39%	34	5.61%
Antepartum eclampsia	5	2.15%	5	0.94%	42	6.93%
Maternal request	4	1.72%	19	3.58%	23	3.80%
Total	233	100%	531	100%	606	100%

## Discussion

This retrospective study showed a significant increase in CS rate over time. Out of 9775 deliveries conducted during the study period, the rate of CS increased from 39.63% in 2011 to 52.42% in 2021, and the rate of vaginal delivery decreased from 60.37% in 2011 to 47.58% in 2021. Another study also showed an increase in the rate of cesarean delivery globally [9]. This significant shift in delivery methods over the past decade reflects a broader global trend towards higher CS rates, which has been attributed to a variety of factors including changes in clinical practices, increased antenatal visits leading to increased detection of high-risk pregnancy, increased maternal age and cases of infertility, precious pregnancy, and recently a significant rise in maternal request for CS delivery [10].

In our study, out of 4644 CS, 62.79% of cases are primiparous, and 37.20% of cases are repeat CS. The rate of primary CS has increased in the last decade [11]. The indication of CS in primiparous should be wisely identified so that the rate of the primary CS could be reduced which in turn will reduce the rate of repeat CS and the future complications of CS.

An analytical study on primary CS at a tertiary hospital in Mysore, India, reported a CS rate of 25.87%. The study concluded that measures such as regular use of partograph, judicious use of amniotomy and oxytocin, and expertise in instrumental vaginal delivery could help reduce CS rates [12]. CS delivery rates in India are higher among highly educated, urban women with more than four antenatal care visits and those from richer families and Christian religions [13]. Another study shows the CS rate in a tertiary care hospital in Kashmir is very high compared to WHO standards, with the previous CS being the commonest indication [14].

A detailed examination of the indications for primary elective CS reveals that oligohydramnios with IUGR remained the most common indication, increasing from 30.97% in 2011 to 34.30% in 2021. Other significant indications include malpresentation and cephalopelvic disproportion. Notably, maternal request as an indication saw a marked increase from 6.19% in 2011 to 15.70% in 2021, highlighting a growing trend in patient-driven decisions for elective CS. For primary emergency CS, fetal distress was consistently the leading indication, although its prevalence decreased from 28.57% in 2011 to 17.45% in 2021. Other common indications included non-progress of labor and antepartum hemorrhage. There was a notable rise in the incidence of severe pre-eclampsia and antepartum eclampsia as indications for emergency CS, reflecting an increased recognition and management of these high-risk conditions. There is a decrease in the incidence of obstructed labor as an indication of CS from 13.17% in 2011 to 7.00% in 2021, but still, its percentage is high reflecting the need to educate professional healthcare providers and traditional birth attendants about the grave complications of obstructed labor and identification and early referral of cases of dysfunctional labor.

In the context of repeat CS, oligohydramnios with IUGR and maternal request emerged as the most frequent indications of elective repeat CS by 2021. The incidence of maternal requests increased significantly from 5.88% in 2011 to 23.91% in 2021, aligning with the trends observed in primary elective CS [15]. This shift indicates a growing patient preference for planned repeat CS. For emergency repeat CS, scar tenderness was the predominant indication throughout the study period, with its prevalence slightly decreasing from 45.76% in 2016 to 42.57% in 2021. Other notable indications included fetal distress and non-progress of labor. There was a significant decline in cases of obstructed labor as an indication for repeat emergency CS, from 6.44% in 2011 to 0% in 2021, possibly reflecting improved labor management techniques and timely decision-making in obstetric care during the trial of labor after CS.

A study at King Fahd Medical City in Riyadh identified increasing maternal age, parity, and prematurity as significant factors associated with CS. Non-reassuring cardiotocography was the most common indication for CS, suggesting that secondary tests for fetal well-being might help reduce CS rates [16]. In North Tanzania, a study found a 26.75% prevalence of CS following labor induction. Risk factors for CS delivery included primiparity, high birth weight, post-term pregnancy, and urban residence. The study emphasized the need for the assessment of these factors before labor induction to reduce adverse outcomes associated with emergency CS [17]. Another study on indications for CS among primigravida in a tertiary care center found that obstructed labor and fetal distress were the main reasons for CS. The study recommended timely and accurate screening during obstetric care and early referral from peripheral hospitals to tertiary centers for a trial of vaginal delivery [18].

While these indications reflect critical clinical decisions, it is important to evaluate the impact of rising CS rates on maternal and neonatal health. Interestingly, despite the increasing frequency of CS, neonatal morbidity and mortality rates have remained constant over the years [19]. According to recent studies, the rate of grave maternal complications like placenta accreta syndrome is on a significant rise, and even neonatal complications such as respiratory distress and infections have not decreased, highlighting a need for further investigation into the efficacy of current obstetric practices [20]. Several factors may contribute to this phenomenon, including the inherent risks associated with surgical deliveries, variations in healthcare quality, and potential overuse of CS in non-critical cases. The increasing reliance on CS has implications beyond immediate neonatal outcomes [21]. It raises concerns about maternal health, healthcare costs, and long-term effects on both mothers and infants. Therefore, it is crucial to adhere to evidence-based guidelines and ensure that CS is performed only when medically necessary. Adequate maternity care, health education, and appropriate training of attending healthcare workers at regular intervals will help in informed decision-making about CS [22]. The use of partographs, increased training in instrumental vaginal delivery, and provision of standardized protocols for elective CS may further reduce CS without suboptimal indication [23,24].

The study has a few limitations. The study is from a single tertiary center relying on retrospective data which may be subject to inaccuracies or incomplete records. This limits the ability to establish causality between the observed trends and the underlying factors. While common indications for CS were documented, the study may not capture all the reasons for the increase in CS rates. The study lacks detailed data on maternal outcomes and long-term health impacts on both mothers and infants, which are critical for evaluating the full implications of rising CS rates. Addressing these limitations in future research could provide a more comprehensive understanding of the factors driving the increase in CS rates and help develop strategies to optimize delivery practices.

## Conclusions

The study indicates a rising trend in CS rates at tertiary care centers, with various clinical and non-clinical factors influencing this increase. The increasing rates of maternal requests for CS, both primary and repeat, point towards a shift in patient preferences and expectations. Additionally, the changing trends in indications for both elective and emergency CS highlight evolving clinical practices and an increased emphasis on maternal and fetal safety. Reducing unnecessary CS is important because it helps minimize the risks for both the mother and baby. Continuous monitoring and analysis of these trends are essential to ensure that the indications for CS are appropriately managed, balancing the benefits and risks associated with surgical deliveries and the provision of evidence-based maternity care to the parturient.
